# Remote Ischemic Preconditioning Prevents Acute Kidney Injury Following Coronary Angiography: The BRICK Randomized Clinical Trial

**DOI:** 10.1016/j.jacadv.2025.102092

**Published:** 2025-08-23

**Authors:** Oladipupo Olafiranye, Adam C. Straub, Yingze Zhang, Rahul Chaudhary, Megan P. Miller, Kathan Trivedi, Lawrence Hoang, Oussama Khalifa, Adetola Ladejobi, Floyd W. Thoma, Ning Feng, Aref Rahman, John A. Kellum, BRICK Trial Investigators

**Affiliations:** a Division of Cardiology, Department of Medicine, UT Southwestern Medical Center, Dallas, Texas, USA; b Division of Cardiology, Department of Medicine, Veterans Affairs North Texas Health Care System, Dallas, Texas, USA; c Department of Pharmacology and Chemical Biology, University of Pittsburgh School of Medicine, Pittsburgh, Pennsylvania, USA; d Heart, Lung, Blood and Vascular Medicine Institute, University of Pittsburgh School of Medicine, Pittsburgh, Pennsylvania, USA; e University of Pittsburgh Medical Center, Pittsburgh, Pennsylvania, USA; f Division of Cardiology, Department of Medicine, Veterans Affairs Pittsburgh Healthcare System, Pittsburgh, Pennsylvania, USA; g Department of Medicine, Dallas Methodist Medical Center, Dallas, Texas, USA; h Department of Cardiology, Memorial Healthcare System, Hollywood, Florida, USA; i Center for Critical Care Nephrology, University of Pittsburgh, Pittsburgh, Pennsylvania, USA

**Keywords:** cardiac catheterization, contrast-induced nephropathy, ischemic conditioning

## Abstract

**Background:**

Patients undergoing invasive coronary angiography have an increased risk of acute kidney injury (AKI) for which there is no well-defined prophylactic therapy.

**Objectives:**

This study examined whether remote ischemic preconditioning (RIPC) reduces the rate of AKI in high-risk patients undergoing invasive coronary angiography.

**Methods:**

In this randomized sham-controlled trial, we enrolled high-risk patients undergoing coronary angiography between March 2018 and February 2023. Patients were randomly assigned 1:1 to RIPC or sham-RIPC. The primary outcome was the rate of AKI after coronary angiography. Secondary endpoints included changes in vascular and urinary biomarkers after RIPC, major adverse cardiovascular and cerebrovascular events (MACCE), and major adverse kidney events at 6-month follow-up.

**Results:**

A total of 109 patients (median age, 75 years) were randomized to RIPC (n = 54) and sham-RIPC (n = 55). The rate of AKI was lower in the RIPC compared to the sham-RIPC group (14.8% vs 29.1%; OR: 0.43; 95% CI: [0.17-0.94]; *P* = 0.030). The product of urinary tissue inhibitor of metalloproteinase-2 and insulin-like growth factor-binding protein-7, 2 markers of AKI, significantly increased at 48 hours post-angiography compared to pre-angiography level (0.51 vs 0.89; *P* = 0.004) in the sham-RIPC but not in the RIPC group (0.39 vs 0.68; *P* = 0.091). At 6-month follow-up, RIPC reduced the rate of MACCE (16.7% vs 36.4%; OR: 0.35; 95% CI: [0.14-0.87]; *P* = 0.029) but not major adverse kidney events (7.4% vs 10.4%; OR: 0.65; 95% CI: [0.17-2.46]; *P* = 0.740).

**Conclusions:**

In high-risk patients undergoing coronary angiography, RIPC reduced incidence of AKI and MACCE, and positively influenced biomarkers of kidney injury. (Biochemical Effects of Remote Ischemic Pre-Conditioning on Contrast-induced Acute Kidney Injury [BRICK]; NCT03236441)

Acute kidney injury (AKI) is common among patients with acute coronary syndrome (ACS) undergoing coronary angiography and percutaneous coronary intervention (PCI), and it is associated with high morbidity and mortality.^1^, ^2^, ^3^, ^4^, ^5^, ^6^, ^7^ The incidence of AKI is estimated to be about 5% to 50% in patients undergoing coronary angiography with higher rates in patients with acute myocardial infarction undergoing PCI.^1^^,^^2^ In particular, elderly patients with multiple comorbidities undergoing PCI have a high risk of developing AKI.^8^^,^^9^ Guidelines emphasize AKI prevention strategies before coronary angiography in these high-risk patients.^10^^,^^11^

Remote ischemic preconditioning (RIPC), elicited by brief episodes of ischemia and reperfusion at a distant vascular bed, has shown promise for protection of vital organs from ischemic injury.^12^, ^13^, ^14^ Some studies indicated that RIPC may attenuate AKI associated with coronary angiography,^15^ PCI,^16^, ^17^, ^18^ and cardiac surgery.^19^, ^20^, ^21^ Among patients with acute ST-segment elevation myocardial infarction (STEMI), Olafiranye et al. previously demonstrated that RIPC administered during emergency helicopter transport decreased incidence of AKI following primary PCI.^17^ However, some trials have yielded negative results.^22^^,^^23^ Whether or not RIPC has renoprotective effects in ACS patients undergoing coronary angiography remains controversial. Hence, we conducted a two-center randomized controlled trial in a high-risk cohort of patients with ACS to assess the effects of RIPC on AKI and clinical outcomes, as well as to elucidate the mechanistic underpinnings of these effects using urinary and serum biomarkers.

## Methods

### Study design

This 2-center, randomized, double-blinded, sham-controlled clinical trial was conducted at the University of Pittsburgh Medical Center and the affiliated Veterans Affairs Pittsburgh Healthcare System in the United States. The protocol of the BRICK (Biochemical and Reno-Protective Effects of Remote Ischemic Preconditioning on Contrast-Induced Kidney Disease) trial including sample size determination and statistical plan are available in the Supplemental Appendix. This study followed the Consolidated Standards of Reporting Trials as detailed in the Supplemental Appendix. Informed consent was obtained from all eligible patients, and the study was approved by the Institutional Review Board.

### Patient population

**Figure 1:**
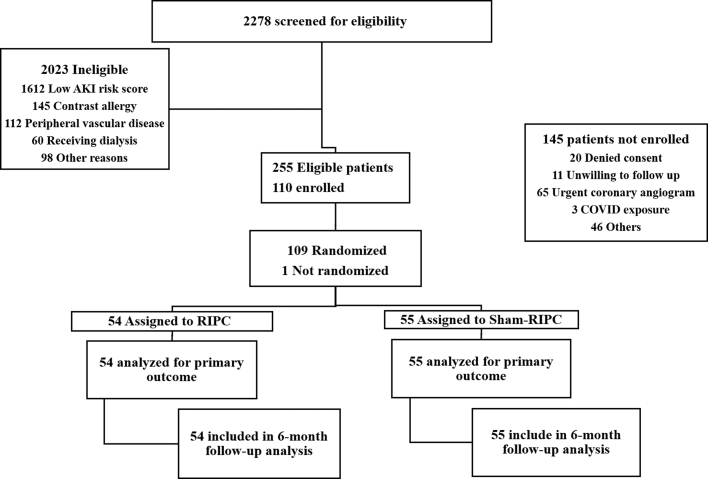
**Flowchart of Screening, Randomization, and Follow-Up of the Patients** AKI = acute kidney injury; RIPC = remote ischemic preconditioning.

Adult patients over the age of 18 years with ACS (unstable angina, non-STEMI) at high risk for AKI, as determined by the modified Mehran AKI risk score of ≥11, who were receiving coronary angiography and/or PCI were eligible. The Mehran risk score assigns points for age >75, heart failure, diabetes, hypotension, intra-aortic balloon pump use, low glomerular filtration rate, and total contrast volume. A score of ≥11 corresponds to ≥26% risk of AKI as previously described.^9^ For preprocedure risk stratification, we modified the original Mehran score by presuming a contrast volume of <100 mL (which assigns 1 point) during the initial calculation, as the actual contrast volume would only be determined after completion of the procedure. This modification allowed us to identify high-risk patients before exposure to contrast media, while still maintaining the predictive validity of the Mehran score for AKI risk assessment. Unstable angina was defined as chest pain or discomfort caused by an insufficient flow of blood and oxygen to the heart that occurred with minimal exertion or at rest with negative cardiac enzymes. NSTEMI was defined as chest pain or discomfort with positive cardiac enzymes but without new ST-segment elevation or left bundle branch block on electrocardiogram. Patients with inability to give informed consent, STEMI, unstable blood pressure (systolic blood pressure >200 or <90 mm Hg), peripheral vascular disease, contrast allergy, or renal disease requiring dialysis and/or placement of arteriovenous fistula and graft were excluded. We enrolled a total of 110 patients between March 2018 and February 2023. Enrollment was impacted by COVID-19 pandemic in 2020 to 2021. As shown in Figure 1, of the 110 patients enrolled, 1 patient withdrew after signing informed consent and before randomization, and the remaining 109 patients were randomized in 1:1 ratio to RIPC (N = 54) and sham-RIPC (N = 55). Baseline comorbidity data were obtained through review of electronic medical records and subsequent cross-verification with the patient at the time of enrollment.

### Randomization and blinding

Eligible patients were randomized to RIPC or sham-RIPC immediately after obtaining informed consent. The randomization was computer-generated using Research Electronic Data Capture data management application with predefined variable allocation block sizes of 2 to 4, and stratification by site. Access to the randomization page of Research Electronic Data Capture was password-protected and only granted to unblinded research staff who performed randomization and delivery of RIPC and sham-RIPC. Blinding of patients was ensured through the use of sham-RIPC in the control group. The investigators, cardiologists performing coronary angiography, outcome assessors, and data analysts were blinded to RIPC allocation throughout the trial.

### Procedures

Patients randomized to RIPC underwent 3 cycles of 5-minute upper arm ischemia using a manual blood pressure cuff placed around the upper arm inflated to 200 mm Hg and interrupted 3 times for 5-minute reperfusion with the cuff deflated (total of 30 minutes) within 4 hours before coronary angiography. Patients randomized to the control group received a sham-RIPC similar to the RIPC protocol, but rather than the cuff being inflated to 200 mm Hg to induce transient limb ischemia, the cuff was instead inflated to a nonocclusive 10 mm Hg to provide the impression of a treatment being administered. This intervention was carried out concurrently with usual care for coronary artery disease with no interruption as much as possible in both treatment and control groups. In accordance with the standard clinical practice, all patients were permitted to receive intravenous (IV) fluid for hydration. The total IV fluid volume, as indicated in Table 1, denotes the total volume of IV fluids given during the perioperative period, including preprocedure, intraprocedure, and up to 24 hours postprocedure. Oral fluid intake was not systematically tracked in this trial and was not accounted for in the total volume of fluid.

**Table 1:** Baseline Demographic, Clinical and Angiographic Characteristics

	Sham-RIPC (n = 55)	RIPC (n = 54)	*P* Value
Age, y	75.0 (71.0-79.5)	76.0 (71.0-81.8)	0.680
Race			
White	92.7% (51)	92.6% (50)	1.000
Black	7.3% (4)	7.4% (4)	
Gender			
Men	85.5% (47)	83.3% (45)	
Women	14.5% (8)	16.7% (9)	0.970
Medical history			
Hypertension	89.1% (49)	79.6% (43)	0.270
Dyslipidemia	89.1% (49)	74.1% (40)	0.080
Diabetes	74.5% (41)	81.5% (44)	0.520
Prior myocardial infarction	29.1% (16)	35.2% (19)	0.630
Prior heart failure	74.5% (41)	79.6% (43)	0.690
Prior CABG	27.3% (15)	22.2% (12)	0.700
Aspirin use	63.6% (35)	70.4% (38)	0.590
Clopidogrel use	20.0% (11)	29.6% (16)	0.350
Prasugrel use	0.0%	0.0%	1.000
Ticagrelor use	5.5% (3)	1.9% (1)	0.620
Beta blockers use	70.9% (39)	70.4% (38)	1.000
ACEi Use	27.3% (15)	33.3% (18)	0.630
ARB use	27.3% (15)	27.8% (15)	1.000
Statin use	72.7% (40)	66.7% (36)	0.630
Diuretic use	52.7% (29)	37.0% (20)	0.150
Clinical presentation			
Unstable angina	65.5% (36)	61.1% (33)	0.780
NSTEMI	34.5% (19)	38.9% (21)	
Procedural outcomes. angiographic and laboratory findings			
PCI	54.5% (25)	64.8% (19)	0.370
CABG	5.5% (3)	5.6% (3)	1.000
Left main (≥50%)	16.4% (9)	11.1% (6)	0.600
Proximal LAD (≥70%)	36.4% (20)	35.2% (19)	1.000
Proximal circumflex (≥70%)	34.5% (19)	27.8% (15)	0.580
Proximal RCA (≥70%)	25.5% (14)	18.5% (10)	0.520
LVEF (%)	50.0 (30.0-55.0)	45.0 (40.0-55.0)	0.900
Creatinine, baseline (mg/dL)	1.2 (1.0-1.6)	1.3 (0.9-1.6)	0.820
eGFR, baseline (mL/min/1.73 m^2^)	62.1 (22.3)	59.7 (23.9)	0.580
BUN, baseline (mg/dL)	29.0 (25.0-37.8)	26.0 (20.0-47.5)	0.320
Total IV fluid volume (mL)	557.6 (656.5)	486.3 (395.7)	0.510
Total contrast (mL)	62.5 (50.0-110.0)	77.5 (45.0-147.5)	0.386
Modified Mehran risk score^a^	13.0 (12.0-15.0)	14.0 (12.0-15.0)	0.350
Mehran risk score^a^	13.0 (12.0-14.5)	13.0 (12.0-15.0)	0.200

Values are median (IQR) or mean (SD). Categorical variables presented as n (%).

ACEi = angiotensin converting enzyme inhibitor; ARB = angiotensin receptor blocker; BUN = blood urea nitrogen; CABG = coronary artery bypass graft; eGFR = estimated glomerular filtrate rate; IV = intravenous; LAD = left anterior descending; LVEF = left ventricular ejection fraction; NSTEMI = non-ST elevation myocardial infarction; PCI = percutaneous coronary intervention; RCA = right coronary artery; RIPC = remote ischemic preconditioning.

a Modified Mehran risk score refers to the preprocedure risk assessment where a contrast volume of <100 mL (1 point) was assumed for patient selection preprocedure. Mehran risk score represents the final calculated score using the actual contrast volumes administered during the procedure.

### Study outcomes

The primary study outcome was the development of AKI, defined according to the 2012 KDIGO guidelines as either an absolute increase in serum creatinine of ≥0.3 mg/dL or a relative ≥50% increase within 48 hours after coronary angiography compared with the pre-angiography creatinine value.^24^

Secondary outcomes included the changes in the product of urinary tissue inhibitor of metalloproteinases-2 (TIMP-2) and insulin-like growth factor-binding protein 7 (IGFBP7), (TIMP-2) x (IGFBP7), and plasma cyclic guanylate monophosphate (cGMP). Other secondary clinical outcomes were the incidence of: 1) major adverse cardiovascular and cerebrovascular events (MACCE) including rehospitalization for myocardial infarction, repeat revascularization, hospitalization for heart failure, stroke, and cardiac death; and 2) major adverse kidney events (MAKE) including initiation of dialysis and all-cause death at 6 months.

### Biomarkers analysis

#### Serum creatinine

The diagnosis of AKI is traditionally based on a rise in serum creatinine, a chemical waste product produced by the muscles, and that serves as a rapidly mobilizable reserve of high-energy phosphates in skeletal muscle. Serum creatinine was determined from samples collected pre-angiography (baseline), as well as 24 hours and 48 hours post-angiography for the assessment of AKI. These assays were performed at the Translational Research Core Laboratory at the University of Pittsburgh. The baseline estimated glomerular filtration rate was calculated using the 2009 Chronic Kidney Disease Epidemiology Collaboration equation at the initial phase of the study,^25^ whereas 2021 Chronic Kidney Disease Epidemiology Collaboration equation was used for the latter phase.^26^

#### Urinary insulin-like growth factor-binding protein 7 and tissue inhibitor of metalloproteinases 2

TIMP-2 and IGFBP7, both inducers of G1 cell cycle arrest, are implicated in AKI and serve as biomarkers to predict it. The product of urinary TIMP-2 and IGFBP7 concentrations, (TIMP-2) × (IGFBP7), was measured with the NEPHROCHECK (R) test (Astute 140 Meter; bioMérieux France). We measured urinary TIMP-2 × IGFBP7 from samples obtained at baseline, immediately after 3 cycles of RIPC but before coronary angiography, and at 24 hours and 48 hours after coronary angiography. These assays were performed at Center for Critical Care Nephrology core laboratory at the University of Pittsburgh according to the manufacturer’s specification as previously described.^19^

#### Plasma cyclic guanylate monophosphate

A well-defined target for nitric oxide (NO) is serum guanylate cyclase (sGC) which is activated through a specific interaction with NO. Once activated, sGC catalyzes the conversion of guanosine 5′-triphosphate to cGMP. The subsequent rise in cGMP concentration is what allows sGC to transmit an NO signal to the downstream elements of the signaling cascade, particularly in the smooth muscle cells, leading to a decrease in Ca^2+^ concentration and ultimately causing smooth muscle relaxation and vasodilatation, as proposed as one of the protective effects of RIPC.^14^^,^^27^^,^^28^ Plasma concentration of cGMP was measured using blood samples obtained at baseline, immediately after 3 cycles of RIPC but before coronary angiography, and at 24 hours and 48 hours after coronary angiography. These assays were performed at the Center for Microvascular Research laboratory at the University of Pittsburgh using the standard enzyme immunoassay kit from Cell Signaling (Cat. #4360) as previously described.^29^^,^^30^

### Statistical analysis

We calculated sample size based on the primary endpoint with intention to show prevention of AKI by RIPC in high-risk cardiac patients. We considered the incidence rate for AKI of 36% to 40% and absolute risk reduction (ARR) of 26% to 28% as reported in trials performed in patients at a high risk for AKI.^16^^,^^18^ We then estimated that a sample size of 100 patients (50 RIPC and 50 sham-RIPC) would provide 88% power to detect a 25% ARR in the RIPC group (ie, incidence of AKI is 35% in controls and 10% in RIPC group). An additional 10 patients were recruited to account for loss to follow-up or nonevaluative data.

The primary efficacy analysis included all randomized patients (full analysis set) and was performed according to the intent-to-treat principle, that is, all patients were analyzed according to their randomization. For the primary outcome, a 1-sided Z-test was used to assess the hypothesized reduction in AKI rates in light of the pre-established directionality of the effect of RIPC. For all secondary outcomes, 2-sided tests were used. All reported CIs are conventional 2-sided 95% CIs. Patients discharged before 48 hours were required to return as outpatient for blood and urine sample collection. AKI status was assigned based on the last known AKI status at 48 hours post-coronary angiography. For secondary clinical outcomes and other categorical variables, descriptive statistics were summarized as frequency (%) and compared between groups using a chi-square test or Fisher exact test if any cell had an expected count of <5. Continuous variables, expressed as mean ± SD, were compared between groups with an unpaired *t*-test. Continuous variables that were not distributed normally were expressed as median (IQR), and analyzed with nonparametric tests (Mann-Whitney *U* and Wilcoxon for unpaired and paired observations, respectively). We estimated the RR reduction and the ARR, including 95% CIs, for the occurrence of AKI, comparing the 2 study cohorts. For the secondary biomarker variables, a *t*-test analysis was conducted to assess the variations in mean renal biomarker levels across distinct time points. For biomarker analyses, we performed both within-group comparisons using paired *t*-tests for comparisons of the changes in biomarker levels. For between-group analyses, we calculated the change in biomarker levels between time points (pre-RIPC to post-RIPC, pre-RIPC to 24 hours, and pre-RIPC to 48 hours) for each subject and compared these changes between the RIPC and sham-RIPC groups using independent *t*-tests and Mann-Whitney *U* tests. No adjustments for multiple comparisons were performed given the modest sample size. All statistical analyses were performed using SPSS (version 26.0).

## Results

The demographics of the study are displayed in Table 1. The median age of our cohort was 75 years (IQR: 67-83 years); patients were mostly male (85%) and White (92%). Of the 109 patients who were randomized, 54 received RIPC and 55 received sham-RIPC. As summarized in Table 1, the RIPC and sham-RIPC groups have similar demographic and baseline clinical characteristics. Both groups exhibit similar prevalence rates of hypertension, dyslipidemia, and diabetes.

Rates of unstable angina (unstable angina: 65.5% in sham-RIPC; 61.1% in RIPC) and NSTEMI (32.7% vs 38.9%) clinical presentation were similar in both groups. Similar rates of PCI (54% in sham-RIPC; 64% in RIPC) and coronary artery bypass graft (5.5% in sham-RIPC; 5.6% in RIPC) were also observed in the 2 groups. In addition, total fluid received, left ventricular ejection fraction, total contrast volume, AKI risk score, baseline creatinine, and blood urea nitrogen levels were similar between both groups (Table 1).

The rate of AKI was significantly lower in the RIPC (8/54) compared to the sham-RIPC (16/55) group (14.8% vs 29.1%; OR: 0.43; 95% CI: 0.17-0.94; *P* = 0.030) with approximately 50% RR reduction by RIPC. As shown in Table 2, at the 6-month follow-up, RIPC was associated with significantly lower rate of MACCE (16.7% vs 36.4%; OR: 0.35; 95% CI: 0.14-0.87; *P* = 0.029) but not MAKE (7.4% vs 10.4%; OR: 0.65; 95% CI: 0.17-2.46; *P* = 0.53). For the individual components of MACCE and MAKE, RIPC was associated with lower rate of hospitalization for heart failure (7.4% vs 21.8%; OR: 0.35; 95% CI: 0.09-0.95; *P* = 0.05), but there was no significant difference in other clinical outcomes as shown in Table 2.

**Table 2:** Clinical Outcomes at the 6-Month Follow-Up

Outcomes	Sham-RIPC (n = 55)	RIPC (n = 54)	Risk Difference (95% CI)	*P* Value
Major adverse cardiac and cerebrovascular events	20 (36.4)	9 (16.7)	−19.7% (−35.8 to −3.6)	0.029
Rehospitalization for myocardial infarction	6 (10.9)	2 (3.7)	−7.2% (−16.9 to 2.5)	0.270
Hospitalization for heart failure	12 (21.8)	4 (7.4)	−14.4% (−27.4 to −1.5)	0.049
Repeat coronary revascularization	6 (10.9)	4 (7.4)	−3.5% (−14.3 to 7.3)	0.740
Stroke	2 (3.6)	0 (0)	−3.6% (−8.6 to 1.3)	0.500
Cardiac death	3 (5.5)	0 (0)	−5.5% (−11.5 to 0.5)	0.240
Major adverse kidney events	6 (10.9)	4 (7.4)	−3.5% (−14.3 to 7.3)	0.740
Initiation of dialysis	2 (3.6)	1 (1.9)	−1.8% (−7.9 to 4.3)	1.000
All-cause death	4 (7.3)	4 (7.4)	0.1% (−9.7 to 9.9)	1.000

Values are n (%).

Abbreviation as in Table 1.

**Figure 2:**
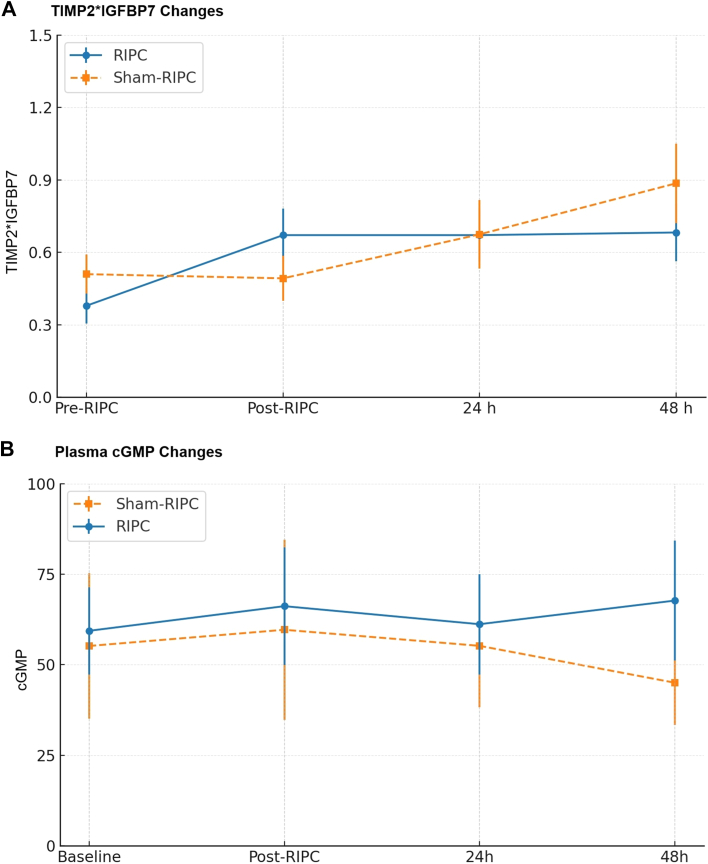
**Pattern of Changes in (TIMP2) x (IGFBP7) and Plasma cGMP** (A) Pattern of changes in mean (SD) levels of the product of urinary tissue inhibitor of metalloproteinases 2 and insulin-like growth factor-binding protein (TIMP2) x (IGFBP7) and (B) plasma cyclic guanylate monophosphate (cGMP) level at different time points in patients who received remote ischemic preconditioning (RIPC) and those who received sham-RIPC. In Figure 2A, (TIMP2) x (IGFBP7) significantly increased from pre-RIPC (baseline) to post-RIPC and before coronary angiography in the RIPC group (*P* = 0.028) but did not change in the sham-RIPC group (*P* = 0.327). At 48 hours postinvasive coronary angiography, (TIMP2) x (IGFBP7) significantly increased in the sham-RIPC group (*P* = 0.004) but not in the RIPC group (*P* = 0.091) when compared to the pre-RIPC level. In Figure 2B, there were no statistically significant differences in mean levels of cGMP between precoronary and postcoronary angiography time points in either RIPC or Sham-RIPC group.

As shown in Table 3, there were some critical differences in urinary (TIMP-2) × (IGFBP7) levels between various time points in the sham-RIPC and RIPC groups. In the sham-RIPC group, (TIMP-2) × (IGFBP7) significantly increased from pre-RIPC to 48 hours (0.51 vs 0.89; *P* = 0.004). Whereas in the RIPC group, the different between pre-RIPC and 48 hours was less (0.39 vs 0.68) and did not reach statistical significance, (*P* = 0.091). Importantly, as expected in the RIPC group, there was an initial significant rise in (TIMP-2) × (IGFBP7) from pre-RIPC to post-RIPC and before coronary angiography (0.39 vs 0.71; *P* = 0.028) whereas no such change occurred in the sham-RIPC group (0.51 vs 0.49; *P* = 0.619) as shown in Figure 2A. Although the pattern of change in cGMP levels suggests an initial rise following RIPC and before coronary angiography, there were no statistically significant differences between pre-coronary and post-coronary angiography point in either RIPC or sham-RIPC groups. Between-group comparisons of the changes in biomarker levels from pre-RIPC to subsequent time points showed no statistically significant differences between RIPC and sham-RIPC groups. For TIMP-2 × IGFBP7, the RIPC group showed mean increases from baseline to 24 and 48 hours (+0.23 and + 0.31, respectively), whereas the sham-RIPC group showed mean decreases (−0.10 and −0.07, respectively). For cGMP, both groups showed increases from baseline with numerically larger but nonsignificant increases in the RIPC group compared to sham-RIPC at all time points (Central Illustration).

**Table 3:** Comparison of Levels of Urinary TIMP2 x IGFBP7 and cGMP at Different Time Points

	Comparison	T-Stat	*P* Value	Baseline	Post-RIPC	24 Hours	48 Hours	Mean Difference (95% CI)
Within-group comparisons: (TIMP-2) × (IGFBP7) biomarker								
Sham-RIPC	Pre-RIPC vs Post-RIPC	0.502	0.619	0.51 (0.51)	0.49 (0.56)			−0.02 (−0.13 to 0.17)
Sham-RIPC	Pre-RIPC vs 24 h	−1.332	0.193	0.51 (0.51)		0.67 (0.89)		0.16 (−0.18 to 0.51)
Sham-RIPC	Pre-RIPC vs 48 h	−3.247	0.004	0.51 (0.51)			0.89 (0.84)	0.38 (0.13–0.63)
RIPC	Pre-RIPC vs Post-RIPC	0.978	0.028	0.39 (0.51)	0.71 (1.58)			0.32 (0.04–0.61)
RIPC	Pre-RIPC vs 24 h	−1.922	0.063	0.39 (0.51)		0.67 (0.66)		0.28 (−0.02 to 0.58)
RIPC	Pre-RIPC vs 48 h	−1.749	0.091	0.39 (0.51)			0.68 (0.66)	0.29 (−0.01 to 0.59)
Within-group comparisons: cGMP levels								
Sham-RIPC	Pre-RIPC vs Post-RIPC	−0.990	0.327	55.19 (147.46)	59.66 (181.04)			4.47 (−3.83 to 12.77)
Sham-RIPC	Pre-RIPC vs 24 h	0.017	0.987	55.19 (147.46)		55.22 (116.02)		0.03 (−8.36 to 8.42)
Sham-RIPC	Pre-RIPC vs 48 h	−1.358	0.185	55.19 (147.46)			45.04 (65.00)	−10.15 (−22.51 to 2.21)
RIPC	Pre-RIPC vs Post-RIPC	−1.646	0.106	60.12 (84.71)	68.28 (112.56)			8.16 (−0.54 to 16.86)
RIPC	Pre-RIPC vs 24 h	−2.599	0.130	60.12 (84.71)		62.91 (89.36)		2.79 (−5.19 to 10.77)
RIPC	Pre-RIPC vs 48 h	−1.675	0.104	60.12 (84.71)			65.61 (96.01)	5.49 (−3.48 to 14.46)
Between-group comparisons: (TIMP-2) × (IGFBP7) biomarker								
Between groups	Pre-RIPC to Post-RIPC Change	−0.271	0.787	Mean change: Sham-RIPC = −0.02 (0.29); RIPC = −0.001 (0.40)				
Between groups	Pre-RIPC to 24 h Change	−0.989	0.326	Mean change: Sham-RIPC = −0.10 (1.88); RIPC = +0.23 (0.60)				
Between groups	Pre-RIPC to 48 h Change	−0.915	0.364	Mean change: Sham-RIPC = −0.07 (2.09); RIPC = +0.31 (0.78)				
Between-group comparisons: cGMP levels								
Between groups	Pre-RIPC to Post-RIPC Change	−0.478	0.634	Mean change: Sham-RIPC = +5.34 (38.92); RIPC = +9.39 (45.66)				
Between groups	Pre-RIPC to 48 h Change	−0.352	0.726	Mean change: Sham-RIPC = +8.06 (32.50); RIPC = +11.22 (37.28)				

Values are mean (SD) unless otherwise indicated.

(TIMP-2) × (IGFBP7) = the product of urinary tissue inhibitor of metalloproteinases 2 and insulin-like growth factor-binding protein 7; cGMP = plasma cyclic guanylate monophosphate; other abbreviation as Table 1.

**Central Illustration:**
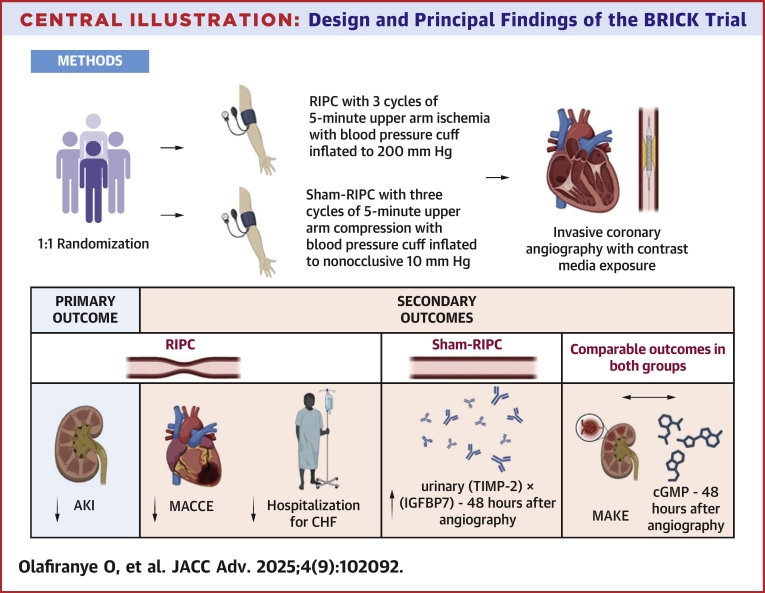
**Design and Principal Findings of the BRICK Trial** The top part shows the randomization of patients into RIPC and sham-RIPC groups before invasive coronary angiography. The bottom part shows the changes in the primary and secondary outcomes in the RIPC and sham RIPC groups. (TIMP-2) x (IGFBP7) = the product of urinary tissue inhibitor of metalloproteinases 2 and insulin-like growth factor-binding protein 7; AKI = acute kidney injury; cGMP = plasma cyclic guanylate monophosphate; CHF = clinical heart failure; MACCE = major adverse cardiovascular and cerebrovascular events; MAKE = major adverse kidney events; RIPC = remote ischemic preconditioning.

## Discussion

The major finding of this first U.S.-based randomized controlled trial on RIPC in ACS patients undergoing invasive coronary angiography is that RIPC has the potential to modify outcomes in high-risk elderly patients undergoing invasive coronary angiography. Our finding of a significant reduction in AKI rate by RIPC with ∼50% RR reduction demonstrates the efficacy of RIPC as a protective strategy against AKI in this vulnerable patient population. This finding not only aligns with the hypothesized benefit of RIPC but also extends previous evidence from other studies where similar protective effects against AKI were observed.^31^ These results suggest that implementing RIPC in clinical practice could potentially improve outcomes for high-risk patients undergoing coronary angiography by reducing the incidence of AKI, a complication associated with significant morbidity and health care costs.^3^^,^^4^

Although the mechanisms of kidney protection by RIPC are not entirely clear, potential pathophysiological mechanisms involve complex humoral signaling pathways.^14^ These include release of NO and adenosine, release of damage-associated molecular pattern molecules, mitigation of free radicals, and modulation of mitochondrial activity. These processes ultimately enhance the ability of the kidney to withstand prolonged ischemia and reperfusion injury.^14^^,^^19^ The pattern of change in urinary (TIMP-2) × (IGFBP7) by RIPC before coronary angiography is consistent with the proposed mechanism of the effect of RIPC on “alarm” markers. Zarbock et al previously showed that increases in (TIMP-2) × (IGFBP7) in response to RIPC protect the kidney from AKI.^19^ This alarm marker sends a signal of danger, causing a temporary induction of G1 cell-cycle arrest, an epithelial defense mechanism in the renal tubules.^19^ Similar results were found in a more recent trial in China evaluating the effect of delayed RIPC on AKI in patients undergoing coronary artery bypass graft surgery.^32^ In line with this theory, we observed a significant elevation in urinary (TIMP-2) × (IGFBP7) following RIPC and before coronary angiography with no further change after coronary angiography. In contrast, in the sham-RIPC, there was no change in the urinary level of (TIMP-2) × (IGFBP7) before coronary angiography; however, following the procedure, a significant increase in urinary (TIMP-2) × (IGFBP7) was observed up to 48 hrs indicating kidney stress and a higher risk of AKI in this group after exposure to contrast media during invasive coronary angiography.

This finding is important because TIMP-2 and IGFBP7 are much more sensitive to kidney injury than changes in serum creatinine which require enough damage to result in functional impairment. Recently, Husain-Sayed et al found that in patients undergoing cardiac surgery without AKI by KDIGO criteria, but with postoperative increases in TIMP-2 and IGFBP7 experienced a similar decrease of renal functional reserve with clinically apparent AKI.^33^ Given that renal tubular toxicity is one of the key pathophysiological concepts of AKI following contrast media exposure, it stands to reason that RIPC may prevent contrast-related AKI via (TIMP-2) × (IGFBP7)-mediated temporary cell cycle arrest and increased resistance of the renal tubular epithelial cells to the toxic effect of contrast media at the time of exposure.^28^ In addition, RIPC may counteract contrast-induced vasoconstriction of renal arteries via vasodilation induced by the release of endogenous nitrite and cGMP. Although not statistically significant, the pattern of change in cGMP in the current study suggests an initial numerical rise following RIPC and before coronary angiography. Although the between-group comparisons of changes in biomarker levels did not reach statistical significance, likely due to high individual variability, the observed patterns are consistent with our proposed mechanism for RIPC's protective effects.

Another finding of the present study is an association of RIPC with reduced incidence of MACCE which was partly driven by lower rates of hospitalization for heart failure. This finding is consistent with the reports from the LIPSIA conditioning trial involving STEMI patients in Germany.^34^ In LIPSIA trial, the combination of RIPC and postconditioning elicited by intracoronary balloon inflations was found to reduce major adverse cardiac event (MACE) consisting of cardiac death, reinfarction, and new congestive heart failure.^34^ Among STEMI patients undergoing primary PCI, our group showed an association of RIPC with a lower rate of clinical heart failure symptoms and serum brain natriuretic peptide level.^35^ Although the mechanism of the effect of RIPC on clinical heart failure is not completely understood, it may operate through multiple pathways beyond the vasodilatory activity of RIPC-induced endogenous nitrite.^14^^,^^27^^,^^28^ The prevention of AKI itself could mediate the reduction in heart failure hospitalizations through several potential mechanisms: 1) AKI directly contributes to volume overload which may precipitate heart failure exacerbations; 2) AKI without adequate recovery can lead to reduced kidney function and impaired volume regulation, increasing heart failure decompensation risk; and 3) medication discontinuation practices following AKI—particularly diuretic agents and renin-angiotensin-aldosterone system inhibitors—may lead to suboptimal heart failure management and increased risk of decompensation.^36^ These hypothetical theories necessitate rigorous investigation in larger clinical trials to ascertain the degree to which AKI prevention mediates the beneficial effects of RIPC on heart failure hospitalizations.

Although Yamanaka et al found no difference in MACE with RIPC compared to the control at 30 days after PCI in the acute myocardial infarction population in Japan,^18^ long-term follow-up data from a similar population in Denmark revealed a reduction in MACE and all-cause mortality in patients who received RIPC compared to those who did not.^37^ Interestingly, our finding of significant reduction in AKI by RIPC did not lead to a reduction in MAKE at 6 months. This is due in part to the low rate of persistent renal dysfunction requiring dialysis in the population studied. Overall, 3 patients required dialysis; 2 in the sham-RIPC group and 1 in the RIPC group.

### Study limitations

First, the study population was predominantly older patients, with 90% being above the age of 65 years. So, the results may not be generalizable to younger adults. Second, our sample size was modest; however, the study was enhanced by the inclusion of patients at a high risk for AKI using the modified Mehran risk score. For the primary analysis, we performed a 1-tailed Z test, as the sample size was only powered to detect a decrease, not an increase, in the AKI rate by RIPC. Third, our statistical analyses did not incorporate adjustments for multiple comparisons; however, the baseline characteristics were well balanced between the RIPC and sham-RIPC groups for the primary analyses. The notable associations observed between RIPC and secondary outcomes should be considered hypothesis-generating and require validation in larger multicenter studies. Finally, extended creatinine measurements beyond 48 hours were not systematically collected in this trial. Future studies should consider incorporating a 7-day KDIGO timeframe to enhance detection of AKI. Despite these limitations, the trial has some important virtues. First, it builds on prior studies and provides further evidence of the potential beneficial effect of RIPC on kidney injury and MACCE in the growing elderly population. Second, it suggests potential clinical benefits on heart failure that warrant further investigation.

## Conclusions

In high-risk elderly patients undergoing coronary angiography, RIPC reduced the incidence of AKI and MACCE, and positively influenced biomarkers of kidney injury. Larger clinical studies are needed to confirm the relationship between RIPC and MACCE and to explore its effects on other clinical outcomes, particularly clinical heart failure.

Perspectives **COMPETENCY IN MEDICAL KNOWLEDGE:** In this BRICK randomized clinical trial, RIPC reduced the rate of AKI and MACCE in high-risk patients with ACS. Biomarkers of kidney injury were positively impacted by RIPC.

**TRANSLATIONAL OUTLOOK:** RIPC has a potential to reduce the risk of AKI and adverse events in high-risk patients undergoing coronary angiography. Larger clinical studies are needed to confirm the relationship, and to explore the effects of RIPC on clinical heart failure.

## Funding support and author disclosures

The BRICK trial was funded by the National Institute of Diabetes Digestive and Kidney Disease grant R21DK113486 (O. Olafiranye). Dr Olafiranye is supported by research grants from the United States Veterans Affairs Clinical Science Research and Development Merit Award (I01 CX002045) and bioMérieux France NEPHROCHECK (R) award (MTA00010106). Dr Kellum has received consulting fees from bioMérieux and is an inventor on a patent concerning the use of TIMP2 x IGFBP7 with RIPC. The contents are solely the responsibility of the authors and do not necessarily represent the official views of the funding agencies. All other authors have reported that they have no relationships relevant to the contents of this paper to disclose.
