# Supplementary material for: Remote Ischemic Preconditioning Prevents Acute Kidney Injury Following Coronary Angiography: The BRICK Randomized Clinical Trial
Source: JACC Adv. 2025 Aug 23;4(9):102092. doi: 10.1016/j.jacadv.2025.102092 (PMC12397924; doi:10.1016/j.jacadv.2025.102092)
Supplement: Supplemental Data [file mmc1.pdf]

Clinical Trial Protocol

Biochemical and Reno-Protective Effects of Remote Ischemic Preconditioning on Contrast-Induced Kidney Disease (BRICK) Trial

Version	Date	Main reason for changes
1	12/20/2017	Not applicable
2	02/26/2019	Addition of a second trial site

1. ***Background***

Acute kidney injury (AKI) is a common complication of intravenous, iodinated contrast media, that is widely used for cardiac catheterization and percutaneous coronary intervention (PCI) in patients with coronary artery disease (CAD).[1,2] In the United States, CAD remains the number one cause of death in both men and women despite improvement in the care of patients over the last decade. Although PCI restores blood flow to the heart, the contrast media used for the procedure can cause AKI, possibly mediated by contrast–induced vasoconstriction of renal blood vessels and free radical–mediated direct renal tubular toxicity. The incidence of AKI is estimated to range between 10 and 40% in patients undergoing cardiac catheterization with higher rates in patients with acute myocardial infarction.[1,2] In the United States, approximately 1.4 million cardiac catheterization procedures are performed each year, and this estimate is expected to increase exponentially in the next few decades. With increasing use of contrast media, the prevalence of AKI is also expected to rise. AKI predicts elevated risk of heart attack, longer in-hospital stay, more complicated hospitalization course, and higher in-hospital mortality. Unfortunately, there is no effective prophylactic regimen to prevent AKI.

Remote ischemic pre-conditioning (RIPC), elicited by application of one or more brief episodes of ischemia and reperfusion of a limb, is a promising therapy for preventing or attenuating AKI. Given that renal ischemic injury and tubular toxicity are the most common pathophysiological concepts of AKI, it stands to reason that RIPC may prevent AKI via nitrite-induced vasodilation and damage associated molecular protein -mediated renal cell protection. Our preliminary data suggest that RIPC provides renal protection and indicate a connection between RIPC-induced changes in protective molecules (nitrite, cyclic guanosine

monophosphate [cGMP)], tissue inhibitor of metalloproteinases 2 [TIMP-2], and insulin-like growth factor–binding protein 7 [IGFBP7] and organ protection. However, the effect of RIPC on AKI in patients with CAD undergoing cardiac catheterization is not well-established, and the underlying mechanism of such effect remains unclear.

2. *Study Design and Objective*

This is a prospective, double-blinded, two-center, randomized, sham-controlled clinical trial. The primary aim of this study is to determine whether remote ischemic preconditioning (RIPC) reduces the incidence of acute kidney injury (AKI) in high-risk patients undergoing coronary angiography and/or PCI. The secondary aim is to study the effect of RIPC on renal (TIMP-2 X IGFBP7) and vascular (cGMP) biomarkers, major adverse cardiac events (MACE), and major adverse kidney events (MAKE) during a 6-month follow-up period in high-risk patients undergoing coronary angiography.

3. *Patient*

Eligible male and female patients over the age of 18 years with unstable angina or non-ST elevation myocardial infarction (NSTEMI) who are at high risk for AKI and undergoing coronary angiography and/or PCI. The trial will be conducted at two tertiary hospitals in the United States, the University of Pittsburgh Medical Center and the affiliated Veterans Affairs Pittsburgh Healthcare System. AKI risk will be determined by a modified version of Mehran's risk score. The scoring system contains different risk factors, including patient characteristics, comorbidities and contrast volume. At the time of enrollment and prior to coronary angiography, the lowest range of contrast volume (1-100) in the scoring system will be utilized for calculation of modified Mehran risk score as shown in Table 1.[3] After coronary angiogram, the actual volume of contrast used during the procedure will be utilized to calculate post-angiography Mehran risk score. A score of 11 or higher will be used to define patients at high risk for AKI as shown in Table 2.[3] Patients with inability to provide informed consent, acute ST elevation myocardial infarction, unstable blood pressure (BP) (systolic BP > 200 or <90 mmHg), peripheral vascular disease, contrast allergy, renal disease requiring dialysis, or placement of arteriovenous fistula graft will be excluded.

Table 1: Scoring System for Predicting Acute Kidney Injury	
Risk Factors	Score
Age > 75	4
Diabetes mellitus	3
NYHA Class III/IV Heart failure	5
Anemia (male: HCT<39, female: HCT<36)	3
Hypotension (SBP<80mmHg or >1hr inotropic support)	5
Intra-aortic balloon pump placement	5
Estimated GFR < 20ml/min	6
Estimated GFR 20-40 ml/min	4
Estimated GFR 40-60ml/min	2
Contrast volume (1-100cc)	1
Contrast volume (101-200cc)	2
Contrast volume (201-300cc)	3
Contrast volume (301-400cc)	4
Contrast volume (401-500cc)	5

Table 2: Risk Score and Predicted Risk of Acute Kidney Injury and Dialysis		
Mehran Risk Score	Risk of AKI	Risk of Dialysis
≤ 5	7.5%	0.04%
6-10	14%	0.12%
11-16	26.1%	1.09%
>16	57.3%	12.6%

Inclusion Criteria

Adult patients over the age of 18 years

Diagnosis of non-ST elevation myocardial infarction or unstable angina

Referral for invasive coronary angiogram and/or percutaneous coronary intervention

Acute kidney injury risk score of ≥11

Exclusion Criteria

Inability to give informed consent

Unstable blood pressure (systolic blood pressure > 200 or <90 mmHg)

History of allergy to contrast media

Peripheral vascular disease of upper limb

Renal disease requiring dialysis

Placement of arteriovenous fistula and arteriovenous graft

4. *Randomization and Blinding*

A total of 110 patients will be randomized to the RIPC (55) treatment group or the Sham-RIPC (55) control group on a 1:1 basis. Randomization will be computer-generated using the REDCap data management application system. Unblinded statistician will set up a pre-defined allocation table with variable block sizes of 2 to 4, and stratification by site. Unblinded research staff will perform randomization and application of RIPC and sham-RIPC. Patients will be blinded by the use of Sham-RIPC in the control group. Investigators, cardiologist performing coronary angiography, clinical outcome assessors, and data analysts will be unaware of treatment assignment.

5. *Procedures, blood and urine sampling and analysis*

All patients will undergo coronary angiography and/or PCI using the standard technique. The decision to perform PCI, choice of guidewires, balloons, stent types, and characteristics will be made by the interventional cardiologist according to common laboratory practice and guideline for coronary angiography and interventions. All patients will receive standard medical therapy and hydration for coronary angiography and PCI. We will extract data on patient demographic and medical history including gender, age, race, comorbidities, and coronary angiography

findings from the electronic medical record.

After randomization, we will perform RIPC or Sham-RIPC approximately 1-4hrs before coronary angiography. The patients assigned to RIPC will undergo 3 cycles of 5-minute inflation of a standard blood-pressure cuff to 200 mmHg, followed by 5-minute cuff deflation. In patients assigned to the control group, sham-RIPC will be induced using 3 cycles of 5-minute blood pressure cuff inflation to a pressure of 10 mmHg, followed by 5-minute cuff deflation.

For the primary aim outcome of AKI, blood samples will be drawn at baseline and then at 24hrs and 48hrs after coronary angiography for measurement of serum creatinine using the standard laboratory protocol. Urinary insulin-like growth factor-binding protein 7 (IGFBP7) and tissue inhibitor of metalloproteinases 2 (TIMP-2), both inducers of G1 cell cycle arrest, are implicated in AKI and serve as biomarkers to predict it. [4] The product of urinary TIMP-2 and IGFBP7 concentrations, (TIMP-2) × (IGFBP7), will be measured with the NephroCheck Test. We will measure urinary TIMP-2 × IGFBP7 from samples obtained at baseline, immediately after three cycles of RIPC but before coronary angiography, 24hrs, and 48hrs after coronary angiography. These assays will be performed at the Center for Critical Care Nephrology core laboratory at the University of Pittsburgh according to the standard manufacturer's specification. Plasma concentration of cyclic guanylate monophosphate (cGMP) will be measured using blood samples obtained at baseline, immediately after 3 cycles of RIPC but before coronary angiography, 24hrs, and 48hrs after coronary angiography. These assays will be performed at the Center for Microvascular Research laboratory at the University of Pittsburgh using the standard enzyme immunoassay kit.

6. Outcomes

The primary end point is the occurrence of AKI, defined as a relative increase in serum creatinine of $\geqslant$ 0.3mg/dl compared with baseline creatinine within 48 hours after coronary angiography according to the Kidney Disease: Improving Global Outcomes guideline.[5] Secondary biomarker endpoints include the product of urinary concentrations of TIMP-2 and IGFBP7 and plasma concentration of cGMP.
Other secondary clinical outcomes are 1) major adverse cardiovascular and

cerebrovascular events (MACCE) including rehospitalization for myocardial infarction, repeat revascularization, hospitalization for heart failure, stroke, and cardiac death and 2) major adverse kidney events (MAKE) including use of renal replacement therapy and all-cause death during the 6-month follow up.

7. *Statistical analysis*

Sample size and power analysis for the primary outcome: Limited data are available for estimation of sample size for the effect of RIPC on incidence of AKI in high-risk patients with unstable angina or NSTEMI undergoing coronary angiography or PCI. Assuming an estimated incidence rate of AKI of 36-40% in high-risk study population as documented in prior studies, and considering absolute risk reduction (ARR) of 26-28% as reported in trials performed in patients at moderate to high risk for AKI,[6,7] a sample size of 100 patients (50 RIPC and 50 Sham-RIPC) will provide 88% power to detect a 25% ARR in the RIPC group (i.e. incidence of AKI is 35% in controls and 10% in RIPC group). Relative powers for different levels of effect sizes for our estimated sample size (N=100) are shown in the Table 1 below. We will enroll 110 patients to allow for up to 10% loss to follow up.

Table 1: Estimated Power with N=100 at Various Effect Sizes

		**AKI Incidence (RIPC Arm)**		
		**10%**	**15%**	**20%**
**AKI Incidence (Sham-RIPC)**	**30%**	*73%*	*44%*	*21%*
	**35%**	*88%*	*65%*	*39%*
	**40%**	*95%*	*81%*	*59%*

Descriptive Statistics: Baseline socio-demographic and clinical characteristics will be summarized using mean and standard deviation when normally distributed, and median and interquartile range when not normally distributed. Categorical variables will be summarized using relative frequencies and percentages.

Primary outcome: the goal is to determine whether RIPC reduces the rate of AKI in high-risk patients undergoing coronary angiography and/or PCI. As this is a categorical variable with an expected reduction in AKI rate, the hypothesis will be tested using risk ratio to determine if the difference in incidence of AKI is significantly lower in RIPC group compared to the Sham-RIPC group. We will explore baseline variables for univariable associations with AKI and examine for independent association of RIPC with AKI using logistic regression. The primary

analyses will be based on intention-to-treat principle.

Secondary outcomes (biomarkers): the goal is to characterize changes in the product of TIMP2 and IGFBP7 and plasma cGMP following RIPC in patients with CAD undergoing coronary angiogram. We will test the association of RIPC with levels of each molecule (RIPC vs. non-RIPC groups) using parametric statistics (t-test, confidence intervals for differences) when levels were normally distributed (expected), or using transformed levels if not. If distributions are very unbalanced, nonparametric tests will be used. The extent of protocol violations and missing outcome data will be quantified overall and by random assignment. All statistical analyses will be performed using SPSS.

8. *Ethics*

The written informed consent form, and overall study protocol and materials will be reviewed and approved by the IRB before the initiation of this trial. A Data and Safety Monitoring Board (DSMB) will be created to review this study. The DSMB will meet after initial approval with plan for quarterly meeting. However, the follow-up meeting frequency of the DSMB will be determined during the first meeting. An emergency meeting of the DSMB will be called at any time by the Chairperson should questions of patient safety arise.

9. *Informed consent*

All study patients will be provided with an easily understandable informed consent form approved by the IRB. The patients will be enrolled after signing the informed consent form. The informed consent form will be kept as an important document of the clinical trials for future reference.

References

1. Tsai TT, Patel UD, Chang TI, Kennedy KF, Masoudi FA, Matheny ME, Kosiborod M, Amin AP, Messenger JC, Rumsfeld JS, Spertus JA. Contemporary incidence, predictors, and outcomes of acute kidney injury in patients undergoing percutaneous coronary interventions: insights from the NCDR Cath-PCI registry. JACC Cardiovasc Interv. 2014 Jan;7(1):1-9.
2. Rihal CS,TextorSC,GrillDE,BergerPB,Ting HH, Best PJ, Singh M, Bell MR, Barsness GW, Mathew V, Garratt KN, Holmes DR Jr: Incidence and prognostic importance of acute renal failure after percutaneous coronary intervention. Circulation 105: 2259–2264, 2002.
3. Mehran R1, Aymong ED, Nikolsky E, Lasic Z, Iakovou I, Fahy M, Mintz GS, Lansky AJ, Moses JW, Stone GW, Leon MB, Dangas G. A simple risk score for prediction of contrast-induced nephropathy after percutaneous coronary intervention: development and initial validation. J Am Coll Cardiol. 2004;44:1393–9.
4. Zarbock A, Schmidt C, Van Aken H, Wempe C, Martens S, Zahn PK, Wolf B, Goebel U, Schwer CI, Rosenberger P, Haeberle H, Görlich D, Kellum JA, Meersch M; RenalRIPC Investigators. Effect of remote ischemic preconditioning on kidney injury among high-risk patients undergoing cardiac surgery: a randomized clinical trial. JAMA. 2015;313(21):2133-41.
5. Kidney Disease: Improving Global Outcomes (KDIGO) Acute Kidney Injury Work Group. KDIGO Clinical Practice Guideline for Acute Kidney Injury. Kidney inter., Suppl. 2012; 2: 1–138.
6. Deftereos S, Giannopoulos G, Tzalamouras V, Raisakis K, Kossyvakis C, Kaoukis A, Panagopoulou V, Karageorgiou S, Avramides D, Toutouzas K, Hahalis G, Pyrgakis V, Manolis AS, Alexopoulos D, Stefanadis C, Cleman MW. Renoprotective effect of remote ischemic post-conditioning by intermittent balloon inflations in patients undergoing percutaneous coronary intervention. J Am Coll Cardiol. 2013 May 14;61(19):1949-55.
7. Yamanaka T, Kawai Y, Miyoshi T, Mima T, Takagaki K, Tsukuda S, Kazatani Y, Nakamura K, Ito H. Remote ischemic preconditioning reduces contrast-induced acute kidney injury in patients with ST-elevation myocardial infarction: a randomized controlled trial. Int J Cardiol. 2015;178:136-41.

CONSORT Harms 2022 integrated into CONSORT 2010 items checklist of information to include when reporting a randomised trial

Section/Topic	Item No	Checklist item	Reported on page No
**Title and abstract**			
	1a	Identification as a randomised trial in the title	1
	1b	Structured summary of trial design, methods, results of outcomes of benefits and harms, and conclusions (for specific guidance see CONSORT for abstracts)	2
**Introduction**			
Background and objectives	2a	Scientific background and explanation of rationale	4
	2b	Specific objectives or hypotheses for outcomes benefits and harms	4
**Methods**			
Trial design	3a	Description of trial design (such as parallel, factorial) including allocation ratio	5
	3b	Important changes to methods after trial commencement (such as eligibility criteria), with reasons	NA
Participants	4a	Eligibility criteria for participants	5
	4b	Settings and locations where the data were collected	5
Interventions	5	The interventions for each group with sufficient details to allow replication, including how and when they were actually administered	6
Outcomes	6a	Completely defined pre-specified primary and secondary outcome measures for both benefits and harms, including how and when they were assessed	7,8
	6b	Any changes to trial outcomes after the trial commenced, with reasons	NA
	6c	Describe if and how non-prespecified outcomes of benefits and harms were identified, including any selection criteria, if applicable	NA
Sample size	7a	How sample size was determined	9,10
	7b	When applicable, explanation of any interim analyses and stopping guidelines	NA

Section/Topic	Item No	Checklist item	Reported on page No
Randomisation:			6
Sequence generation	8a	Method used to generate the random allocation sequence	6
	8b	Type of randomisation; details of any restriction (such as blocking and block size)	6
Allocation concealment mechanism	9	Mechanism used to implement the random allocation sequence (such as sequentially numbered containers), describing any steps taken to conceal the sequence until interventions were assigned	6
Implementation	10	Who generated the random allocation sequence, who enrolled participants, and who assigned participants to interventions	6
Blinding	11a	If done, who was blinded after assignment to interventions (e.g., participants, care providers, those assessing outcomes of benefits and harms) and how	6
	11b	If relevant, description of the similarity of interventions	6
Statistical methods	12a	Statistical methods used to compare groups for primary and secondary outcomes of both benefits and harms	9,10
	12b	Methods for additional analyses, such as subgroup analyses and adjusted analyses	10
**Results**			
Participant flow (a diagram is strongly recommended)	13a	For each group, the numbers of participants who were randomly assigned, received intended treatment, and were analysed for outcomes of benefits and harms	11
	13b	For each group, losses and exclusions after randomisation, together with reasons	NA
Recruitment	14a	Dates defining the periods of recruitment and follow-up for outcomes of benefits and harms	6
	14b	Why the trial ended or was stopped	NA
Baseline data	15	A table showing baseline demographic and clinical characteristics for each group	26
Numbers analysed	16	For each group, number of participants (denominator) included in each analysis and whether the analysis was by original assigned groups and if any exclusions were made	11, 26
Outcomes and estimation	17a	For each primary and secondary outcome of benefits and harms, results for each group, and the estimated effect size and its precision (such as 95% confidence interval)	11, 26, 28, 29
	17a2	For outcomes omitted from the trial report (benefits and harms), provide rationale for not reporting and indicate where the data on omitted outcomes can be accessed	NA
	17b	Presentation of both absolute and relative effect sizes is recommended, for outcomes of benefits and harms	11,12
	17c	Report zero events if no harms were observed	NA
Ancillary analyses	18	Results of any other analyses performed, including subgroup analyses and adjusted analyses, distinguishing pre-specified from exploratory	11,12
Harms	19	All important harms or unintended effects in each group (for specific guidance see CONSORT for harms)	NA

Section/Topic	Item No	Checklist item	Reported on page No
**Discussion**			
Limitations	20	Trial limitations, addressing sources of potential bias related to the approach to collecting or reporting data on harms, imprecision, and, if relevant, multiplicity or selection of analyses	15,16
Generalisability	21	Generalisability (external validity, applicability) of the trial findings	15,16
Interpretation	22	Interpretation consistent with results, balancing benefits and harms, and considering other relevant evidence	15,16
**Other information**			
Registration	23	Registration number and name of trial registry	1
Protocol	24	Where the full trial protocol and other relevant documents can be accessed, including additional data on harms	Supplement 1
Funding	25	Sources of funding and other support (such as supply of drugs), role of funders	1
